# Supplementary material for: Chromothripsis during telomere crisis is independent of NHEJ, and consistent with a replicative origin
Source: Genome Res. 2019 May;29(5):737–49. doi: 10.1101/gr.240705.118 (PMC6499312; doi:10.1101/gr.240705.118)
Supplement: Supplemental Material [file supp_gr.240705.118_Supplemental_file_1.zip › contigs/annotated_contigs/DB50/contig.2.DB50_length_803_mean_cov_4.80448318804.docx]

**DB50_length_803_mean_cov_4.80448318804**

```
GTTACATGAACTCCTGTAATTTAATATGTTAATCTCTCTTGAGAATCAGGAAGTACATATAAAGAAGCTGCAAAGTTATTTTTCTGAAT
 >chr10:56141669-56142112 - E=1e-248
TAATAGGCTAATAAAGAGGCTAAATGAAAGAGGAAGTTTTCCATTTTTATAAAAAACGTCCCTAGAGGTAAATGGCCCTCAATACCTAC

TTCTTAAAGGATGAAGCACAAATCCCAGTATTTAAGGCTGTAAAAGATTGACCAGCCAAGAAATGTGTTGAAAATATCAATAAATATTT

AACTGTGCTAATATTTTTAACACGATTGTGATTGTTTTGATTTTTATTATTCTAAATACAAGAGAACAAGTCTTTTTCAGGAACATACA

TATAGTATTATAGTAGAAATGCCTTTTCTCTTATAGTACTGATTTTGTAGGGAGAAAAATCGAGGGTAAATGCGGTTATCCACT|TTC|
                                                                                     >ch
TAAGGAGAGGGGTATGGGCACAGTTGTTCAGGCAATTCAGAGTGAACACTTAGGTTAATTTTTAAAAAATATGTATATTGCTCCTTGTA
r10:56140649-56141012 - E=2e-206
TCATGTCTGCCTAAGCTTTGCTCTCTTCTCTCTCTCTCCCCTAAATTTTGGCTTAAAGCAAGCTTGTATATGAAAATTTGAGAGGCTTT

TTTTTTTCTTTTTGGCAATACAAATGGACAATTCTAAAGGGGAAAGCAGGCAAGCAAACAAAACTCTTTCAACACACCAGCAGTAGAAA

GATTTTTTGTTGAGTTTCAATCATTGAACTCAGGAGTGAGCTGAGAGCAAGAAAACTATAGATCTTTTCCATGAAAGTTAGAATCTTTA

AATA
```
